# Parental KIR/HLA‐C Profiles and Reproductive Failure: A Phenotype‐Stratified Retrospective Analysis of Couples With Fertility Disorders

**DOI:** 10.1155/ogi/4578815

**Published:** 2026-07-14

**Authors:** Karin Černá, Jana Voborská Neudeckerová, Pavel Otevřel, Dagmar Ivanická, Michal Koucký, Tereza Kovářová, Štěpánka Luxová

**Affiliations:** ^1^ Immunological Department, GENNET, Na Poříčí 26, Prague, Czech Republic; ^2^ Europe IVF International, Evropská 15, Prague, Czech Republic; ^3^ Reprofit International Brno, Hlinky 48, Brno, Czech Republic; ^4^ Department of Gynecology, Obstetrics and Neonatology, General University Hospital and First Faculty of Medicine, Charles University, Apolinářská 18, Prague, Czech Republic, cuni.cz

**Keywords:** HLA-C, KIR receptors, recurrent implantation failure, recurrent pregnancy loss, reproductive immunology, uterine natural killer cells

## Abstract

Interactions between maternal killer‐cell immunoglobulin‐like receptors (KIR) and fetal human leukocyte antigen‐C (HLA‐C) play a key role in implantation and placentation, yet their clinical relevance remains controversial, partly due to heterogeneous phenotyping of reproductive failure. We conducted a retrospective observational study of 73 biologically comparable clinically referred couples undergoing reproductive immunology assessment. Couples involving donor gametes, surrogacy, or multiple male partners were excluded. Clinical phenotypes were classified as isolated recurrent implantation failure (RIF‐only), isolated recurrent pregnancy loss/adverse pregnancy outcome (RPL/APO‐only), combined RIF + RPL/APO, or no reproductive failure. Maternal KIR phenotype (AA vs. Bx) was determined by flow cytometry and genetically confirmed. Maternal and paternal HLA‐C genotypes were classified as C1C1, C1C2, or C2C2. Associations were assessed using univariable, age‐adjusted, and multivariable logistic regression models. Maternal KIR‐AA was present in 53.4% of women and was more frequent in the RIF‐only group than in the RPL/APO‐only group (56.8% vs. 35.3%). In age‐adjusted analysis, KIR‐AA was associated with increased odds of RIF (OR 2.35; *p* = 0.054), but not with RPL/APO. Maternal HLA‐C genotype showed an exploratory signal consistent with implantation outcomes: C1C2 (OR 0.28; *p* = 0.031) and C2C2 (OR 0.10; *p* = 0.002) were protective against RIF compared to C1C1. In multivariable models, maternal C2C2 remained independently protective (aOR 0.08; *p* = 0.0048). Paternal HLA‐C genotype showed no independent associations. In conclusion, immunogenetic effects in reproductive failure are phenotype‐specific and predominantly influence implantation rather than postimplantation pregnancy loss.

**Highlights**
• Maternal KIR‐AA is preferentially associated with implantation‐related failure.
• Female HLA‐C2 confers protection against recurrent implantation failure.
• Immunogenetic effects are phenotype‐specific rather than uniform.
• Paternal HLA‐C does not independently predict reproductive failure.
• Implantation failure and pregnancy loss represent distinct immunogenetic entities.

## 1. Introduction

From an immunological perspective, human reproduction entails a unique biological paradox, as a successful pregnancy requires maternal immune tolerance toward a genetically semiallogeneic fetus. The key site of this finely regulated process is the feto–maternal interface, where direct interactions occur between invasive extravillous trophoblast cells and maternal decidual immune cells. Among these, uterine natural killer (uNK) cells play a central role as the dominant leukocyte population in the endometrium during early pregnancy. The function of uNK cells differs fundamentally from that of cytotoxic natural killer (NK) cells in peripheral blood: uNK cells are characterized by a regulatory, embryo‐protective immunophenotype, and their primary role is not cytotoxicity but rather the production of cytokines, chemokines, and angiogenic factors that regulate trophoblast invasion and spiral artery remodeling. These processes are essential for the establishment of a low‐resistance uteroplacental circulation and for normal placental development.

A critical regulatory mechanism of uNK cell function is the interaction between their receptors of the killer‐cell immunoglobulin‐like receptor (KIR) family and ligands expressed on the surface of trophoblast cells, particularly human leukocyte antigen‐C (HLA‐C) molecules. HLA‐C is the only classic HLA molecule expressed by extravillous trophoblasts, and it exists in two basic functional groups—designated C1 and C2—which are differentiated by a single amino acid at position 80. These differences determine binding to specific inhibitory or activating KIR receptors [1].

Genetic variability in both KIR genes and HLA‐C alleles gives rise to a wide range of possible maternal–fetal combinations. Simply put, maternal KIR genotypes can be divided into the AA haplotype—which contains predominantly inhibitory receptors—and Bx haplotypes (AB or BB)—which include a greater number of activating receptors. Experimental and clinical data have repeatedly shown that the combination of a maternal KIR‐AA haplotype with the presence of fetal HLA‐C2 (particularly of paternal origin) is associated with impaired placentation and an increased risk of adverse reproductive outcomes, including recurrent implantation failure (RIF), recurrent pregnancy loss (RPL), preeclampsia, intrauterine growth restriction, preterm birth, and other adverse pregnancy outcomes (APO) [2–4].

Despite this growing body of evidence, the clinical interpretation of KIR/HLA‐C interactions remains a matter of debate, largely due to heterogeneity in studied cohorts, differences in the definitions of clinical phenotypes, and the lack of a unified approach to diagnosis and treatment. Direct maternal–fetal interactions occur at the decidual interface, but in clinical cohorts, these interactions are often estimated using parental genotypes, which only provide indirect information about the fetal HLA‐C composition.

The aim of the present study is to analyze our own clinically selected cohort of couples undergoing KIR and HLA‐C testing in relation to observed reproductive difficulties, as well as to situate the obtained results within the context of current knowledge in reproductive immunology.

## 2. Materials and Methods

**FIGURE 1:**
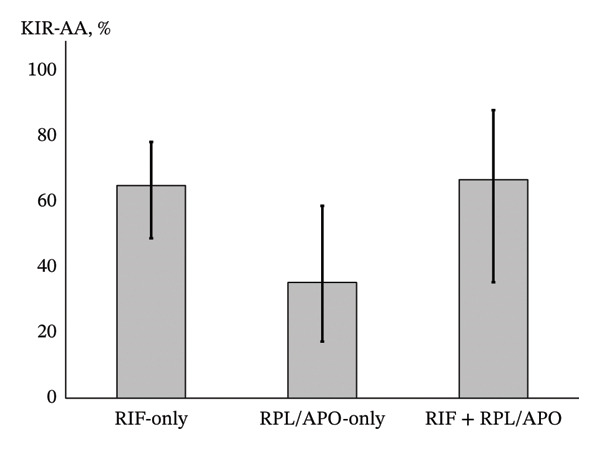
Distribution of maternal KIR haplotypes across clinical reproductive phenotypes. Bars represent the proportion of women with the KIR‐AA haplotype within each clinical subgroup; error bars indicate 95% CIs. KIR‐AA was most frequently observed in RIF‐related phenotypes, whereas no enrichment was observed in pregnancy loss alone.

A total of 120 consecutively referred couples who underwent reproductive immunology testing as part of routine clinical care were initially identified. Prior to inferential analyses, a biologically interpretable analytical cohort was defined to allow valid assessment of maternal–paternal KIR/HLA‐C interactions. Exclusion criteria were based on a priori conceptual considerations regarding fetal genetic origin and interpretability of parental immunogenetic contributions, rather than on study outcomes. Therefore, couples involving donor oocytes or donor embryos (*n* = 14), gestational surrogacy (*n* = 1), or reproductive histories involving multiple male partners with unavailable paternal genotypes (*n* = 32) were excluded. The final primary analytical cohort comprised 73 couples with clearly attributable maternal and paternal genetic contributions (see Figure 1).

The study population was not population‐based. It consisted of clinically referred couples undergoing reproductive immunology assessment because of prior reproductive complications or repeated treatment failure. Immunogenetic testing was performed due to reproductive failure, namely, RIF following in vitro fertilization (IVF) treatment, RPL, other APO, or various combinations of these clinical phenotypes. RIF was classified using a pragmatic clinic‐based definition aligned as closely as possible with recent ESHRE good‐practice recommendations [5]. In general, RIF refers to repeated failure to achieve a clinical pregnancy after the transfer of multiple embryos of appropriate developmental quality in the absence of an obvious anatomical, genetic, or major endocrine cause. Because universally accepted diagnostic thresholds for RIF remain debated, some degree of phenotypic uncertainty is unavoidable). RPL and APO were classified using contemporary guideline‐based definitions; however, retrospective ascertainment may still allow some overlap between categories. RPL was defined according to updated ESHRE recommendations [6] as the loss of two or more clinically recognized pregnancies (only clinically verified pregnancies were included in the analysis). APO was defined as a pregnancy loss and/or preterm delivery between 13 and 34 weeks of gestation, severe preeclampsia requiring delivery before 34 weeks of gestation, or severe intrauterine growth restriction (defined as birth weight below the 10th percentile for gestational age) [7].

In all women, flow cytometric assessment of the KIR receptor haplotype on peripheral blood NK cells was performed according to a previously described methodology [8] and subsequently confirmed by molecular genetic analysis of the KIR system [9]. Patients were classified according to their KIR genotype (based on the presence or absence of individual KIR receptors and genes) into the AA group or the Bx group, with the latter including the AB and BB haplotypes. The fetal HLA‐C genotype was not directly available in this retrospective cohort. Therefore, potential fetal HLA‐C exposure was inferred probabilistically from maternal and paternal genotypes. HLA‐C genotypes were analyzed in women and men and were categorized into three functional groups: C1C1, C1C2, and C2C2 [10].

For clinical interpretability and consistency with prior epidemiological literature, KIR genotypes were categorized as AA versus Bx haplotypes, and HLA‐C genotypes were grouped according to the established C1/C2 epitope system.

### 2.1. Clinical and Laboratory Data Were Evaluated Retrospectively

Statistical analysis was performed using MedCalc Statistical Software (Version 23.1.7; MedCalc Software Ltd., Ostend, Belgium) and R‐based logistic regression routines for multivariable modeling. The distribution of continuous variables was assessed using the Shapiro–Wilk test and was found to deviate from normality; accordingly, nonparametric statistical methods were applied where appropriate. Comparisons of categorical variables were performed using Fisher’s exact test (when expected cell counts were small), or Pearson’s *χ* ^2^ test, where applicable. Continuous variables are presented as medians with interquartile ranges (IQRs), and categorical variables are presented as absolute counts and percentages. One‐sample comparisons with external reference frequencies were interpreted descriptively and were not used as a basis for formal hypothesis testing. Because of the limited sample size and the number of phenotype‐stratified outcomes, the multivariable modeling strategy was simplified in the revised analysis to reduce the risk of overfitting. Events per variable (EPV) were calculated for each logistic regression model. Multivariable models were restricted to clinically prespecified predictors and were interpreted as exploratory. Where EPV was low, results were not considered confirmatory, and emphasis was placed on directionality, effect size, and confidence interval (CI) width rather than statistical significance. Clinical phenotypes were defined a priori and analyzed both in binary outcomes and in phenotype‐specific subgroups, including isolated RIF (RIF‐only), isolated RPL/APO (RPL/APO‐only), and combined phenotypes. Associations between immunogenetic factors (maternal KIR haplotype, maternal and paternal HLA‐C genotypes) and clinical outcomes were evaluated using logistic regression models. Both univariable and multivariable age‐adjusted models were constructed. To explore potential age‐related modifications of immunogenetic effects, additional age‐stratified analyses were performed using predefined age categories (≤ 35 years, 36–39 years, and ≥ 40 years). The results of logistic regression analyses are reported as odds ratios (ORs) or adjusted odds ratios (aORs) with 95% CIs and corresponding *p* values. Given the number of comparisons and the moderate sample size, all inferential analyses were considered exploratory and hypothesis‐generating. No formal family‐wise confirmatory testing framework was prespecified. Therefore, *p* values should be interpreted cautiously and in conjunction with effect sizes, CIs, and biological plausibility. Statistical significance was evaluated at a two‐sided *α* level of 0.05.

## 3. Results

### 3.1. Baseline Descriptive Characteristics of the Clinical Cohort

The cohort comprised a total of 73 heterosexual couples. Basic descriptive characteristics of the cohort are shown in Table 1.

**TABLE 1:** Baseline descriptive characteristics of the study cohort.

Couples evaluated, *n*	73
Female age in years, median (IQR)	37 (33–39)
Clinical phenotype categories, *n* (%)	
RIF‐only	37 (50.7%)
RPL/APO‐only	17 (23.3%)
RIF + RPL/APO	10 (13.7%)
No RIF/RPL/APO	9 (12.3%)
Failed embryo transfers among women with RIF (*n* = 46), median (IQR)	5 (3–6)
Miscarriages at < 12 weeks among women with RPL/APO (*n* = 27), median (IQR)	3 (2–3)
Adverse pregnancy outcomes at > 12 weeks in women with RPL/APO, *n* (%)	12 (46%)

### 3.2. Distribution of KIR Haplotypes in Women

With respect to immunogenetic characteristics, 39 women (53.4%) (95% CI 42.1%–64.4%) exhibited the KIR‐AA haplotype, while the Bx haplotype was present in 34 women (46.6%). The distribution of KIR receptors across different clinical phenotype groups is shown in Figure 1.

### 3.3. Association of Maternal KIR With Different Clinical Phenotypes

The RIF‐only clinical phenotype was more frequent among women carrying the KIR‐AA haplotype than among women with Bx haplotypes. Specifically, KIR‐AA was present in 21 of 37 women with RIF‐only (56.8%), compared with 44.4% of women in the non‐RIF group. This corresponded to numerically higher odds of isolated RIF; however, the estimate was imprecise and not statistically conclusive among KIR‐AA carriers (OR 1.64), although the association did not reach statistical significance in this dataset. In contrast, among women with the RPL/APO‐only clinical phenotype, the KIR‐AA haplotype was not overrepresented, and this corresponds to lower odds of RPL/APO‐only among KIR‐AA carriers (OR 0.68). This finding indicates that KIR‐AA is not associated with an increased risk of pregnancy loss in the absence of implantation failure.

In women with the RIF + RPL/APO phenotype, the KIR‐AA haplotype was observed in 6 of 10 cases—a proportion similar to that observed in controls but higher than that observed in women with the RPL/APO‐only phenotype. Although drawn from low numbers and therefore not statistically robust, this finding suggests that KIR‐AA may contribute to a more severe or complex reproductive phenotype when implantation failure and pregnancy loss co‐occur.

Taken together, these phenotype‐stratified analyses indicate that maternal KIR‐AA does not uniformly increase the risk of adverse reproductive outcomes; rather, it shows distinct and divergent associations across clinical phenotypes (see Table 2).

**TABLE 2:** Association of maternal KIR with clinical reproductive phenotypes.

Clinical phenotype	KIR‐AA, n/N (%)	KIR‐Bx, n/N (%)	Odds ratio (OR)
RIF‐only	21/37 (56.8%)	16/37 (43.2%)	1.64
RPL/APO‐only	6/17 (35.3%)	11/17 (64.7%)	0.68
RIF + RPL/APO	6/10 (60.0%)	3/10 (40.0%)	2.50
no RIF RPL/APO	6/9 (66.6%)	4/9 (33.3%)	reference

### 3.4. Distribution of Female and Male HLA‐C

HLA‐C genotype data were available for all 73 women; the most frequent genotype was C1C1 (45.2%), followed by C1C2 (37.0%) and then C2C2 (17.8%). HLA‐C genotype data for male genetic contributors were also available for all 73 men; the most common genotype was C1C1 (35.6%), followed by C1C2 (34.2%) and then C2C2 (30.1%).

The overall distribution of HLA‐C genotypes did not differ significantly between women and men (*χ* ^2^ test, *p* = 0.200); however, C1C1 was 9.6% more frequent in women than in men (45.2% vs. 35.6%; 95% CI 12.8%–31.0%), whereas C2C2 was 12.3% more frequent in men than in women (30.1% vs. 17.8%; 95% CI 7.3%–30.7%).

### 3.5. Age‐Adjusted and Age‐Stratified Analyses of Immunogenetic Factors

Given the strong and well‐established influence of maternal age on both implantation success and pregnancy maintenance, the associations between immunogenetic factors and reproductive clinical phenotypes were further evaluated by adjusting for female age and using predefined age strata (≤ 35 years, 36–39 years, and ≥ 40 years).

When RIF was analyzed as a binary outcome (RIF present vs. absent), the maternal KIR‐AA haplotype showed directionally higher estimated odds of RIF compared with Bx haplotypes (OR 2.34; 95% CI 0.89–6.19; *p* = 0.053), although the association did not reach conventional statistical significance and should be interpreted with caution. In the unadjusted analysis, KIR‐AA carriers showed higher odds of implantation failure than women with Bx haplotypes (OR 2.34; 95% CI 0.89–6.19; *p* = 0.053). Adjustment for maternal age yielded a similar point estimate, but the CI remained wide and statistical uncertainty persisted. (age‐adjusted OR 2.35; *p* = 0.054). The modest increase in effect size observed after age adjustment is consistent with maternal age acting as a confounder in implantation‐related outcomes (i.e., a well‐established determinant of implantation success). In contrast, no association between maternal KIR‐AA and RPL was identified in either age‐adjusted or age‐stratified analyses, supporting a phenotype‐specific effect that is limited to implantation‐related failure.

Female HLA‐C genotype was also evaluated in age‐adjusted models, using C1C1 as the reference category. For RIF, the presence of the C2 epitope was associated with lower odds of implantation failure: specifically, women with the C1C2 genotype had reduced odds of RIF (OR 0.28; *p* = 0.031), and those with the C2C2 genotype demonstrated an even stronger protective association (OR 0.10; *p* = 0.002). These effects remained directionally consistent across age strata. For RPL/APO, no consistent association with female HLA‐C genotype was observed after adjustment for age. Although a nominally significant association with the C2C2 genotype emerged in one model, this effect was not robust across alternative model specifications or age‐stratified analyses, and the CIs were wide, indicating limited stability of the estimate.

Male HLA‐C genotype was not associated with either RIF or RPL/APO in age‐adjusted analyses. Specifically, neither the C1C2 nor the C2C2 genotype in male genetic contributors showed a meaningful effect on implantation‐related or postimplantation reproductive outcomes, and no age‐dependent modification of these associations was observed.

### 3.6. Multivariable Analyses of Combined Immunogenetic Factors Across Clinical Reproductive Phenotypes

In view of the limited number of events within phenotype‐stratified subgroups, multivariable analyses were simplified and interpreted as exploratory. The number of EPV was below the conventional threshold for stable estimation in several models, particularly for the RPL/APO‐only and combined RIF + RPL/APO phenotypes. Therefore, fully adjusted phenotype‐stratified models were not used as confirmatory evidence.

In the simplified model focused on RIF‐related outcomes, maternal KIR‐AA showed directionally increased odds of RIF, whereas maternal HLA‐C2 carriage showed directionally reduced odds of RIF compared with maternal HLA‐C1C1. However, CIs were wide, indicating limited precision and potential model instability. These findings should, therefore, be regarded as hypothesis‐generating rather than definitive evidence of independent effects.

Because of the low number of events, multivariable models for the RPL/APO‐only and combined RIF + RPL/APO phenotypes were considered underpowered (see Table 3).

**TABLE 3:** Multivariable age‐adjusted logistic regression analyses by clinical phenotype.

Outcome	Events	Predictors included	EPV	Interpretation
RIF present vs. absent	46	maternal age, KIR‐AA, maternal HLA‐C2 carriage	15.3	acceptable for an exploratory model
RIF‐only vs. non‐RIF	37	maternal age, KIR‐AA, maternal HLA‐C2 carriage	12.3	exploratory
RPL/APO‐only vs. non‐RPL/APO	17	maternal age, KIR‐AA only/descriptive only	low	underpowered
RIF + RPL/APO	10	no full multivariable model	very low	descriptive only

## 4. Discussion

The present study analyzes a clinically selected cohort of 73 couples examined within the context of reproductive immunology screening, and it provides several interrelated observations. Notably, the study describes a higher prevalence of the female KIR‐AA haplotype and its possible preferential relationship with implantation‐related phenotypes, although the estimate did not reach conventional statistical significance and requires validation. Our phenotype‐stratified analyses demonstrated that immunogenetic effects do not manifest uniformly across all reproductive failures in clinical practice but instead cluster within specific subgroups, which may help explain the heterogeneity of findings reported in previous studies.

In our cohort, the maternal KIR‐AA haplotype was identified in a substantial proportion of women, at a rate exceeding the frequencies reported in several general European population cohorts of Caucasian ancestry. This finding is consistent with the concept of relative enrichment of an inhibitory KIR‐AA immunogenetic profile among women evaluated for reproductive failure. Although direct one‐sample statistical comparisons with population controls were not the primary aim of the present analysis, the observed prevalence of KIR‐AA in our cohort was numerically higher than that reported in population‐based studies from Spain (24.15%) [11] and Italy (28.5%) [12]. However, it is important to note that the frequency of KIR‐AA observed in our cohort closely parallels that reported in published clinical cohorts of women with recurrent reproductive failure. Notably, the Spanish cohort described by Alecsandru et al. [13], comprising 204 women with RIF and/or RPL, reported a KIR‐AA frequency of 38.7%, which is closer to the prevalence observed in our dataset. This correspondence suggests that the divergence from population‐based reference frequencies is unlikely to reflect random variation and instead may result from clinical selection, with greater referral of women carrying specific immunogenetic profiles to specialized reproductive immunology assessment. Taken together, these observations support the notion that KIR‐AA enrichment is a reproducible feature of clinically affected reproductive cohorts rather than a population‐wide phenomenon, which reinforces the relevance of KIR haplotype assessment in selected clinical contexts.

Among women, the most frequent HLA‐C genotype in our cohort was C1C1 (45.2%), followed by C1C2 (37.0%) and then C2C2 (17.8%). In men, the distribution differed numerically, with C1C1 present in 35.6% of men, C1C2 in 34.2%, and C2C2 at the comparatively high proportion of 30.1% of men. Although the overall distribution of HLA‐C genotypes did not differ statistically significantly between sexes in this small dataset, a numerical enrichment of C1C1 among women and C2C2 among men was consistently observed. This sex‐specific pattern is both biologically noteworthy and in line with established concepts of KIR/HLA‐C interactions at the feto–maternal interface. In reference populations, including a healthy Bulgarian cohort (*n* = 127) [14] and a Caucasian Brazilian population (*n* = 292) [15], the reported HLA‐C genotype frequencies reflect a more balanced distribution of the C1C1, C1C2, and C2C2 genotypes, without marked sex‐specific divergence. These observations suggest that the HLA‐C genotype distribution in a clinically referred reproductive cohort may differ from that of general populations and that such differences may manifest in a sex‐specific manner. Importantly, the observed pattern is compatible with the hypothesis that clinical selection (rather than random sampling) contributes to shaping the immunogenetic landscape of couples presenting with reproductive failure. At the same time, the opposite directions of the deviations in women and in men point away from a simple global shift in HLA‐C frequencies and instead toward distinct selective pressures or referral biases that affect maternal and paternal genotypes differently.

Classic models of reproductive immunogenetics primarily emphasize the axis of the maternal KIR repertoire versus the fetal HLA‐C2 burden, particularly in the context of impaired trophoblast invasion and abnormal placentation. However, a relative excess of male C2C2 genotypes in a clinical cohort may effectively increase the probability of paternal transmission of the C2 epitope to the embryo; consequently, the distribution of fetal C2 burden may be shifted toward higher values than those observed at the population level. This mechanism is fully consistent with placentation models proposed by Moffett’s group [16], which highlight the particular relevance of paternally inherited (allogeneic) HLA‐C2 in shaping uNK cell responses and spiral artery remodeling; it is also in line with experimental and clinical observations indicating that the adverse effects of HLA‐C2 are most pronounced when C2 is of paternal origin.

An unexpected observation of the present study was the inverse association between maternal HLA‐C2 carriage and implantation‐related failure. This finding should be interpreted cautiously, as it does not directly mirror the classical reproductive immunology model emphasizing adverse combinations of maternal inhibitory KIR‐AA with fetal/paternally derived HLA‐C2. Several nonmutually exclusive explanations may account for this discrepancy. First, maternal HLA‐C genotype may reflect a broader immunogenetic background rather than a direct causal effect of maternal HLA‐C molecules themselves. Second, because fetal HLA‐C inheritance was not directly determined, maternal genotype may partially serve as a proxy for the probability of specific maternal–fetal combinations. Third, the present cohort was clinically referred and phenotype‐enriched, which may generate selection patterns different from those in population‐based cohorts. Fourth, the modest sample size and multiple comparisons raise the possibility of chance findings or effect inflation. Therefore, the present result should be considered hypothesis‐generating and not as contradictory evidence against established placentation models. Rather, it may suggest that the maternal genotype context modifies how canonical KIR/HLA‐C mechanisms manifest in selected infertility populations. The present study has several strengths that should be highlighted. One major strength lies in the strict clinical and biological definition of the analyzed cohort, which was refined to include only couples for whom maternal and paternal immunogenetic data could be interpreted within a coherent biological framework. By excluding cases involving donor oocytes, donor embryos, surrogacy, and women with reproductive histories involving multiple partners, we minimized biological heterogeneity and ensured that the analyzed KIR/HLA‐C interactions reflected genuine maternal–paternal–fetal constellations. This approach strengthens the internal validity and distinguishes the present study from broader registry‐based or population‐based analyses. Another important strength is the phenotype‐stratified analytical strategy, which differentiated isolated RIF, isolated RPL/APO, and combined phenotypes. This distinction proved critical, as immunogenetic effects (particularly those related to maternal KIR‐AA) were shown to cluster preferentially in implantation‐related phenotypes rather than across reproductive failure as a single aggregated outcome. This stratification provides a plausible explanation for the heterogeneity of findings reported in earlier studies combining implantation failure and pregnancy loss into a single category. Furthermore, the study benefits from the simultaneous evaluation of maternal KIR genotype and both maternal and paternal HLA‐C genotypes, which allowed the assessment of sex‐specific immunogenetic patterns within the same clinical cohort. The observation that HLA‐C genotype distributions may be shifted in opposite directions in women and men represents a novel contribution and underscores the importance of considering paternal HLA‐C composition when interpreting fetal HLA‐C burden and KIR/HLA‐C interactions.

Nevertheless, several limitations must also be acknowledged. First, the study is retrospective and observational, which precludes causal inference. Although biologically justified, the restriction of the primary cohort may introduce selection effects. Second, despite careful cohort refinement, the sample size was moderate, particularly after phenotype stratification and analysis restriction to complete immunogenetic data. This limitation is reflected in wide CIs for several regression models and reduces the statistical power, especially for the combined RIF + RPL/APO phenotype. Consequently, the multivariable analyses should be interpreted with caution because the sample size was limited relative to the number of predictors and phenotype‐stratified outcomes. Although simplified models were used in the revised analysis, several subgroup models had low EPV, resulting in wide CIs and potential model instability. Therefore, the multivariable findings are presented as exploratory and hypothesis‐generating rather than confirmatory. Third, the analysis relied on simplified categorizations of KIR (AA vs. Bx) and HLA‐C (C1/C2), which, although standard in clinical and epidemiological studies, do not capture the full complexity of the KIR repertoire, gene copy number variation, allelic polymorphism, or functional activity of uNK cells [17]. An important limitation is that the fetal HLA‐C genotype was not directly determined. Because the study relied on parental genotypes, the actual fetal HLA‐C allele inherited in each conception or pregnancy could not be confirmed. Therefore, mechanistic interpretation regarding specific maternal–fetal KIR/HLA‐C combinations remains indirect. The present findings should, thus, be interpreted as the reflecting parental immunogenetic background relevant to maternal–fetal interactions rather than direct evidence of fetal genotype effects. Finally, the cohort represents a clinically referred population rather than a general sample, which limits generalizability to the broader population; however, this characteristic is also intrinsic to the clinical relevance of the study, as the findings are intended to further the understanding of immunogenetic mechanisms in patients with reproductive failure, rather than to estimate population‐level risk. Clinical reproductive phenotypes are biologically complex and may not be fully captured by categorical labels such as RIF or RPL/APO. In particular, RIF lacks universally accepted diagnostic thresholds across the literature. Although contemporary guideline‐based and pragmatic criteria were applied, some degree of phenotype misclassification is possible, especially in retrospective analyses. Such nondifferential misclassification would be expected to attenuate rather than create associations.

## 5. Conclusions

In this clinically referred high‐risk cohort, the observed immunogenetic patterns suggest that reproductive risk may be context‐dependent and influenced by the interaction between maternal KIR repertoire, parental HLA‐C composition, and probable fetal HLA‐C exposure inferred from parental genotypes under consideration. These findings support a simplified population‐level framework that requires validation using higher‐resolution immunogenetic approaches. Future prospective, multicenter studies incorporating larger cohorts, refined phenotyping, and direct assessment of fetal HLA‐C inheritance will be essential for translating these immunogenetic insights into clinically actionable strategies in reproductive medicine.

**Nomenclature**
aOR: Adjusted odds ratio
APO: Adverse pregnancy outcome
CI: Confidence interval
ESHRE: European Society of Human Reproduction and Embryology
HLA‐C: Human leukocyte antigen‐C
IQR: Interquartile range
IVF: In vitro fertilization
KIR: Killer‐cell immunoglobulin‐like receptor
NK cells: Natural killer cells
OR: Odds ratio
RIF: Recurrent implantation failure
RPL: Recurrent pregnancy loss
uNK cells: Uterine natural killer cells

## Author Contributions

Karin Černá, Jana Voborská Neudeckerová, and Dagmar Ivanická designed and supervised the study. Tereza Kovářová and Štěpánka Luxová performed laboratory analysis. Karin Černá wrote the first draft of the paper. Michal Koucký revised the manuscript and added important intellectual content.

## Funding

This research did not receive any specific grant from funding agencies in the public, commercial, or not‐for‐profit sectors.

## Disclosure

All authors approved the manuscript.

## Conflicts of Interest

The authors declare no conflicts of interest.

## Data Availability

The data that support the findings of this study are available on request from the corresponding author. The data are not publicly available due to privacy or ethical restrictions.
